# Applied performance ecology: testing strategies of talent identification in sports using ecological systems

**DOI:** 10.1242/jeb.251395

**Published:** 2026-04-07

**Authors:** Lana A. Waller, Mathew S. Crowther, Simon P. Lailvaux, Frank Seebacher, Robbie S. Wilson

**Affiliations:** ^1^ School of the Environment, The University of Queensland, St Lucia, QLD 4072, Australia; ^2^ School of Life and Environmental Sciences, The University of Sydney, Sydney, NSW 2006, Australia; ^3^ Department of Biological Sciences, University of New Orleans, 2000 Lakeshore Drive, New Orleans, LA 70148, USA

**Keywords:** Ecological models, Individual success, Contests, Crayfish, Traits

## Abstract

Predicting success is a common goal for ecologists and sports scientists, yet these disciplines rarely interact. Sports scientists often use tests of closed-skill or game performances, but these are critiqued for their inherent uncertainties in predicting success. In contrast, ecologists embrace variance, measuring traits under controlled conditions to make probabilistic predictions of success. Integrating ecological perspectives could enhance team selection efficiency in youth sports. Here, we demonstrate this concept using territorial contests in crayfish. As in sports, individual traits in crayfish can be measured rapidly but do not perfectly predict contest outcome. First, we simulated populations of 100 male and 100 female crayfish that competed in 20 rounds of contests and estimated how many individuals must be selected to ensure the top 10% of performers are included. Selections were based on individual traits (body length, claw size and strength) and/or contest outcomes. When few contests had occurred, the top 10% of individuals were most efficiently selected on individual traits but increasingly more on contests as rounds progressed. Empirical data supported these theoretical simulations. We staged 10 rounds of contests among 27 male and 32 female *Cherax destructor*. After two rounds, ∼21 individuals were needed to capture the top 3; by round 10, ∼5 were required. Taken together, our study provides an initial but compelling demonstration of how ecological models can help improve talent identification strategies in sport. Such an adaptive selection framework efficiently narrows down selection of high-performing individuals under uncertainty and has the potential to be applied to reintroduction and translocation strategies in conservation.

## Teaser

**Summary:** Use of a framework informed by ecological models demonstrates how combining trait-based assessments with contest outcomes can improve talent identification in sport by increasing the likelihood of selecting top-performing players, even under uncertainty.

## INTRODUCTION

‘*The important thing in life is not the triumph, but the fight; the essential thing is not to have won, but to have fought well.*’

Pierre de Coubertin – Founder of the Modern Olympics

Ecologists have long studied which traits lead to individual success in nature and how these traits evolve across species and time (Arnold, 1983; Green et al., 2022; Lailvaux and Irschick, 2006; Violle et al., 2007). Just as athletes compete under the rules of their sport, species in nature engage in their own evolutionary contests, shaped by behaviour, environment and ecological constraints (Briffa and Sneddon, 2007; Hardy and Briffa, 2013). In some, success depends on speed; in others, stealth or cooperation. Yet, the goal is the same: to survive and reproduce (Dawkins and Krebs, 1979; Stearns, 1998). In this way, nature resembles a vast arena of overlapping competitive events, each with distinct challenges, strategies and champions. Evolution ‘rewards’ those best suited to their specific game – just as sporting victories go to those most capable under competitive pressure. While nature and sport share these structural parallels, the disciplines that study them – ecology and sports science – have remained surprisingly separate. Both fields grapple with understanding how excellence emerges, is measured and can be predicted, yet there has been limited crosstalk between them. This paper aims to bridge that gap. We argue that conceptual frameworks from ecology – particularly those used to study adaptation and performance – can provide valuable tools for sports science. Specifically, we suggest that sport can be viewed as a form of applied performance ecology, where success is determined by how well individuals meet the demands of complex, competitive environments. To illustrate this perspective, we offer a proof of concept. Drawing from ecological models of territorial competition, we tested an approach for talent identification in competitive team sports using data from animal contests. This dual application demonstrates how ecological thinking can test strategies for decision making in sport.

Talent identification in youth sports aims to recognise individuals most likely to succeed at the professional level. In soccer, expert scouts – relying on match and training observations – remain the dominant evaluators of youth potential (Christensen, 2009; Den Hartigh et al., 2018). However, these assessments are often based on limited observations and are prone to bias and inconsistency (Meylan et al., 2010; Williams and Reilly, 2000). To address this, sports scientists have introduced some structured, more data-informed methods. These often rely on closed-skill tests – controlled assessments of technical abilities isolated from match environments (Ali, 2011; McCalman et al., 2022; Williams and Reilly, 2000) – as well as physical attributes (e.g. body size) and other non-technical performance tests (e.g. sprinting or jumping) (Ali, 2011; Camata et al., 2025; Reilly et al., 2000; Wilson et al., 2025b). When such tests correlate with in-game performance, they can offer valuable predictive information (Phillips et al., 2010; Wilson et al., 2025a). However, critics argue that these tests oversimplify the dynamic nature of match play and typically explain only a modest proportion of variance in complex, game-realistic performances (Bergkamp et al., 2019; Travassos et al., 2013). Thus, players who excel in drills do not always translate their abilities effectively in real matches. Although performance evaluations from game-based assessments may offer richer insights into an individual's ability in matches, these data introduce their own challenges (Heilmann et al., 2022; McCalman et al., 2022). Within games, performance is affected by teammates, opponents and chance (Reilly et al., 2000). Furthermore, it is logistically difficult to run enough games to reliably evaluate all players in large youth cohorts and provide the required statistical confidence on an individual's ability (Wilson et al., 2021). As a result, few professional clubs rely on quantitative approaches alone. Yet, from an ecological perspective, discarding imperfect predictors simply because they do not offer certainty is a missed opportunity. In nature, ecologists routinely measure animal traits under controlled conditions, despite their artificial scenario, because these traits can still explain a proportion of variance of performance in complex environments (Husak et al., 2006; Lailvaux and Husak, 2014, 2017).

Predictive accuracy in ecology often depends on recognising uncertainty and integrating multiple sources of information (Hendry, 2023; Milner-Gulland and Shea, 2017; Mouquet et al., 2015). Performance traits never provide perfect predictions, but they can still improve decision making when used appropriately. For ecologists, success lies in balancing what can be measured with what can be reliably inferred, managing risk by minimising false positives while still capturing signals of promise. Recent work by Wilson et al. (2025b) applied this approach to sport. They developed a data-driven model to identify top performers in youth soccer based on outcomes from 1v1 contests – a simplified, yet dynamic version of real matches. By combining a closed-skill measure (dribbling speed) with win/loss data from over 1300 1v1 games, they showed that initial predictions could be made from closed-skill tests and refined over time with contest outcomes. This adaptive selection model efficiently reduced the pool of players for further scouting while managing uncertainty throughout the process. Their approach mirrored ecological principles: use imperfect traits to guide early decisions and improve predictions through ongoing competitive evaluation.

Beyond offering conceptual tools, ecology can also provide real-world systems to test and refine talent identification strategies. Many animals engage in contests over resources such as mates or territories, often using signals and displays to predict performance (Abalos et al., 2024; Hardy and Briffa, 2013; Maynard Smith and Harper, 2003; Walter et al., 2011). For example, male red deer roar to intimidate rivals (Clutton-Brock and Albon, 1979), while paper wasps use black facial patterns as signals of status to assess rivals before combat (Tibbetts and Dale, 2004; Tibbetts and Lindsay, 2008). In these systems, outcomes of the actual contest are clear (win or loss), and traits associated with contest outcomes, such as size or strength, can be measured rapidly. Crayfish are especially well suited to this kind of analysis. These freshwater crustaceans engage in ritualised contests using their claws, which serve both as signals of strength and as weapons for combat (Bywater et al., 2008; Graham and Angilletta, 2020; Walter et al., 2011; Wilson et al., 2007). When claw size differs, contests are often resolved through visual display alone; when opponents appear evenly matched, strength usually determines the winner (Wilson et al., 2007). Crucially, claw size does not always predict strength, as some individuals have large but weak claws (Walter et al., 2011; Wilson et al., 2007). This mismatch between signal size and true ability mirrors a key challenge in sport: easily measurable traits are not always reliable indicators of competitive success and there are multiple factors that ultimately dictate success (Bergkamp et al., 2019; Vaeyens et al., 2007; Williams and Reilly, 2000; Wilson et al., 2017). Thus, crayfish offer a natural proof of concept for testing talent identification models using ecological systems. Their contests are observable, outcomes are binary and traits can be measured quickly and non-invasively. By integrating trait data with contest outcomes, researchers can build and test predictive models just as one would in sport, while accounting for uncertainty and variance. Crayfish therefore provide an ecologically valid, tractable system for advancing strategies for predicting ecological success and which in this case is analogous to talent identification in sport.

Building on this logic, we used crayfish as a proof of concept to test the adaptive selection model for talent identification proposed by Wilson et al. (2025b). Our goal was to assess how efficiently different selection strategies could identify the top 10% of individuals within a population, based on either traits or contest outcomes, or a combination of the two. We first conducted simulations to estimate the minimum number of individuals that would need to be selected to ensure with 95% confidence that the true top 10% were always selected. Using populations of 100 individuals, we simulated 20 rounds of contests for both males and females, generating a new population for each of 20 replicate simulations. Relationships between body length and claw size and strength were simulated (with variance) using unpublished data (L..A.W. and R.S.W.) drawn from male and female *Cherax destructor*. Each individual was assigned a true dominance value that determined their actual rank in the performance hierarchy, with contest outcomes reflecting these underlying abilities. Contest ability was modelled using a Bradley–Terry model (Bradley and Terry, 1952), and we systematically varied the relative weighting of trait and contest information using an alpha (α) parameter to assess how optimal selection strategies shift as contest data accumulate. We hypothesised that in early rounds, when contest data are sparse, trait-based selection (body length, claw size or claw strength) would be more efficient, requiring fewer individuals to reliably include the top performers. As the contest data set increased, however, we expected contest outcomes to gain increasing predictive power, improving selection precision. To mimic more realistic constraints common in youth sports, we also simulated a second scenario where only the top 40 individuals by trait ranking were allowed to compete in contests. We predicted that this pre-filtering step would further improve efficiency by focusing resources on likely high performers. Finally, we tested these hypotheses using empirical data from staged contests in 27 male and 32 female *C. destructor*. We measured morphological traits (body length, claw size), claw strength and recorded outcomes across 10 rounds of 1v1 contests. Individual dominance scores were estimated using the Bradley–Terry model, and we tracked how the number of individuals that needed to be selected to include the top performers (i.e. the top three by dominance score) changed as contest data accumulated. Consistent with our simulations, we expected the number of required individuals to decline as contest data accumulated, demonstrating the practical value of an adaptive, ecologically grounded selection strategy. By applying this framework to both simulated and empirical crayfish contests, we extend the model of Wilson et al. (2025b) to an ecological system and demonstrate how adaptive selection strategies that integrate multiple information sources while accounting for variance can efficiently identify top performers.

## MATERIALS AND METHODS

### Simulating traits and true dominance

We simulated traits that predicted dominance for 100 male and 100 female crayfish, *Cherax destructor* (Clark 1936), and we repeated these simulations 20 times per sex. Trait equations were based on empirical data describing morphology and claw strength in *C. destructor* (*n*=71 males, *n*=74 females) (see Supplementary Materials and Methods, Eqns S1–S8; L.A.W. and R.S.W., unpublished data). For both sexes, body length was drawn from a normal distribution (mean=0, s.d.=1). Claw size was generated as a linear function of body length with added Gaussian noise (males s.d.=0.26, females s.d.=0.22). Claw strength was derived from claw size with additional noise (males s.d.=0.54, females s.d.=0.69).

We defined a trait-based predictor of contest success for each individual using the combination of traits that best explained outcome in empirical staged contests, as identified by a Bradley–Terry model (L.A.W. and R.S.W., unpublished data). The Bradley–Terry model estimates the probability of an individual winning a contest based on their traits and/or contest outcomes (Bradley and Terry, 1952). We used this model in preliminary analyses to determine which of body length, claw size and claw strength – traits previously shown to influence contest outcome in crayfish (Bywater et al., 2008; Graham et al., 2020; Walter et al., 2011; Wilson et al., 2007) – were most predictive of success in empirical staged contests among *C. destructor* crayfish (as detailed below). For males, the predictor was calculated as an equally weighted sum of claw size and claw strength. For females, the predictor included body length and claw strength. Individuals were then ranked within each sex and replicate according to their trait score (see Table 1 for definitions).

**Table 1.** Definitions of rank types used in simulated and empirical analyses

Rank type	Simulation definition	Empirical definition
Trait rank	Ranking based on sum of simulated traits (e.g. body length, claw size and/or claw strength), each with equal weighting	Ranking based on sum of measured traits (e.g. body length, claw size and/or claw strength), each with equal weighting
Contest rank	Ranking derived from simulated contest outcomes using Bradley–Terry model	Ranking derived from staged contest outcomes using Bradley–Terry model
True rank	Ranking based on simulated true dominance value, calculated by adding noise to trait predictor	Final ranking based on estimated dominance scores from Bradley–Terry model after all contest rounds

An individual's true dominance was simulated by adding noise (s.d.=0.7) to the trait-based predictor (*y*=*x*+error) to reflect variation in competitive ability. Individuals were then ranked within each replicate according to their simulated dominance values to generate their true rank (Table 1).

### Simulating contests and dominance ranks

We simulated 20 rounds of contests per replicate. In each round, 50 pairs of individuals were formed randomly without replacement. Contest outcomes were resolved deterministically such that the individual with the higher true dominance value always won. We recorded the winner and loser of each contest. To estimate dominance abilities from contest outcomes, we fitted a Bradle–Terry model every two rounds (i.e. rounds 2, 4…20), where each model used cumulative contest outcomes up to and including the respective round. Estimated dominance scores for each individual were extracted from the Bradley–Terry models at each respective round and individuals were ranked accordingly to generate contest-based ranks (Table 1).

### Evaluating selection strategies using trait, contest and hybrid ranks

We aimed to quantify how effectively different selection strategies could identify the top 10% of individuals in a simulated population. Each individual was assigned three ranks: a true rank based on their simulated true dominance value, representing their underlying ability; a trait rank based only on measurable traits, calculated as the sum of claw size and claw strength for males or body length and claw strength for females; and a contest rank, reflecting performance in staged contests which was estimated using Bradley–Terry models fitted to cumulative contest outcomes every two rounds. To compare selection strategies, we calculated how many of the top-ranked individuals needed to be selected to ensure that all of the true top 10% (i.e. top 10 based on true dominance) were included. These strategies included selection based on trait ranks only, contest performance only, and a hybrid score combining the two:
(1)

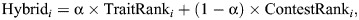
where α (alpha) controls the weighting of trait versus contest information, ranging from 0 (contest only) to 1 (trait only), in increments of 0.1.

We calculated the hybrid score under two scenarios: (i) contests involving all 100 individuals, and (ii) contests conducted after a preliminary cut, where the bottom 60 individuals based on trait rank were removed, such that contests only occurred amongst crayfish with the top 40 trait ranks. For each scenario, contest round and α value, we were therefore able to compare the minimum number of individuals required to ensure the true top 10 were captured.

### Empirical data

#### Study animals and experimental design

We used *C. destructor* sourced from a commercial aquaculture facility (AustSilvers, Swan Bay, NSW, Australia). Individuals were housed at a laboratory at The University of Queensland (St Lucia, QLD, Australia) in individual plastic containers (18×17×18 cm) within larger tanks (61×30×38 cm), at a density of 3–4 crayfish per tank. Tanks contained dechlorinated water, a 2 cm base of gravel and a box filter. Crayfish were fed twice per week with commercial crayfish food pellets [Aqua One Vege Wafers, Kong's (Aust.) Pty Ltd, Ingleburn, NSW, Australia] and were maintained in the laboratory for 12 months before use in the study. We selected 32 females and 27 males that were intermoult and had intact claws for use in experiments. Morphology and claw strength were measured on the first 2 days, followed by staged 1v1 contests that were conducted over a 2 week period.

#### Morphology

We measured the body length and claw size of each crayfish using photographs taken with a digital camera (Casio EXLIM EX-100F, Casio Computer Co. Ltd, Tokyo, Japan), with a scale object included for calibration. Images were analysed using ImageJ (Schneider et al., 2012). Body length was measured as the distance between the tip of the rostrum and the end of the telson. We took seven measurements to quantify claw size, which included the width of the propodus at the carpus and dactyl joints, the width of the pollex and dactylus and the length of the propodus, pollex and dactylus (Bywater et al., 2008; Walter et al., 2011). We used a principal components analysis (PCA) to combine the seven highly correlated claw dimensions into new orthogonal variables, with separate analyses completed for the left and right claws. The first principal component (PC1) was used as a measure of claw size as all claw dimensions were loaded in the same direction, and it explained over 74% of the variation in the claw data. As both claws were approximately symmetrical, we used the mean PC1 score from the left and right claws as the measure of an individual's claw size.

#### Claw strength

We measured the claw strength of crayfish using a custom-built force transducer, as described in detail in Wilson et al. (2007). The transducer consisted of two outer metal plates that were separated by a central metal plate, with a strain gauge attached to the outer side of one plate. The strain gauge measured the change in resistance when the metal plates bent as force was applied by the claws, which was measured via a Wheatstone bridge connected to a BridgePod amplifier (ADInstruments, Bella Vista, NSW, Australia) and recorded using PowerLab software (ADInstruments). The strain gauge was calibrated using known weights to convert the millivoltage output to newtons. We induced crayfish to grab the metal plates at a standardised location and obtained three measures per claw for each individual, with the process repeated the following day. We used the mean of the maximum force generated by the left and right claws as a measure of an individual's maximum claw strength (Bywater et al., 2008; Walter et al., 2011; Wilson et al., 2007).

#### Contests

We staged contests between pairs of same-sex crayfish (male contests *n*=122; female contests *n*=162; Fig. S1). Contests occurred in a 70 l tank (61×30×38 cm) containing gravel and dechlorinated tap water at 25°C. In each round, two randomly selected crayfish were simultaneously placed into the arena and allowed to freely interact (Walter et al., 2011; Wilson et al., 2007). Interactions between two crayfish either involved only signalling (use of antennae and/or tapping of claws) or escalated to fights (grappling and pushing). Contests ended when one crayfish retreated without re-engaging, and this individual was determined as the loser of each pairing. Individuals that lost a claw were excluded from subsequent contests. Ten rounds of contests were conducted, where each individual participated in 8.9±2.2 contests (mean±s.d.).

An eleventh round of contests was conducted in which individuals were paired according to their preliminary ranks after round 10, estimated by a Bradley–Terry model (e.g. rank 1 versus rank 2, rank 3 versus rank 4). This additional round enabled us to improve the reliability of the contest-based rankings. We then estimated each individual's contest rank using all contest outcomes up to and including this final round, which we used as the ‘true’ dominance rank to enable comparison with simulated data (Table 1). As in the simulations, we calculated the minimum number of crayfish that needed to be selected to ensure inclusion of the top 10% (i.e. top 3) based on trait and contest ranks.

#### Statistical analyses

To estimate individual dominance abilities from pairwise contest outcomes, we fitted maximum-likelihood Bradley–Terry models using the *BTm* function from the *BradleyTerry2* package in R (Turner and Firth, 2012). For the simulated data, we fitted models every two rounds (rounds 2–20 in steps of 2) using cumulative contest data up to and including each round. The response variable was the win–loss outcome of each contest, with the probability of individual *i* beating individual *j* modelled as a logistic function of the difference in their abilities. We extracted individual dominance estimates from the models using the *BTabilities* function and converted these to rankings. Separate models were fitted for each sex and replicate.

To examine the relationship between the number of rounds of contests and the number of individuals needed to be selected to include the true top 10% in the simulations, we fitted generalised additive models (GAMs) using the *gam* function from the *mgcv* package in R (Wood, 2017). The GAM modelled the number of individuals needed to include the top 10% as a smooth function of the number of contest rounds, using a basis dimension of *k*=10 to avoid overfitting. Models were fitted using restricted maximum likelihood (REML) as it provides a more robust and stable estimation of smoothing parameters (Wood, 2011, 2017). Rounds 2–20 in steps of two were analysed to improve model fit. Model diagnostics and fit were assessed using the *gam.check* function. Separate models were fitted for males and females.

We fitted additional GAMs to evaluate how different selection strategies (by hybrid score) influence the number of individuals required to ensure inclusion of the top 10%. A tensor product spline (*te* function) was used to model a potential non-linear interaction between α (0 to 0.1 in steps of 0.1) and round (4–20 in steps of 2). Smoothing parameters were estimated using REML, with basis dimensions set to *k*=10 for α and *k*=8 for round. Model diagnostics and fit were assessed as above. Separate models were fitted for males and females, and for the two selection scenarios: (i) when all 100 individuals were included and (ii) when contests after the bottom 60 individuals were cut based on trait rank.

We examined the empirical contest data to assess how well traits predicted dominance. Contest rankings were estimated using Bradley–Terry models, fitted at every second round (rounds 2–10, and 11) using pairwise contest outcomes, following the same methods used in the simulations. We first tested the association between contest rank and the composite trait score, calculated as an equally weighted sum of claw size and strength for males, and of body length and claw strength for females, reflecting the weighting used in the simulations. We fitted a linear regression model using the *lm* function with contest rank as the response variable and trait score as the predictor variable.

To determine the actual relative contribution of each trait to dominance in the empirical data, we fitted additional multiple regression models with claw size and claw strength (males) and body length and claw strength (females) as predictors, with true rank as the response variable. All traits were standardised (mean=0, s.d.=1).

All simulations and analyses were conducted in R version 4.4.3 (https://www.r-project.org/). We used *dplyr* (https://CRAN.R-project.org/package=dplyr) and *tidyr* (https://CRAN.R-project.org/package=tidyr) for data manipulation and *ggplot2* (Wickham, 2016) for data visualisation.

## RESULTS

### Simulated selection across rounds

**Fig. 1.**
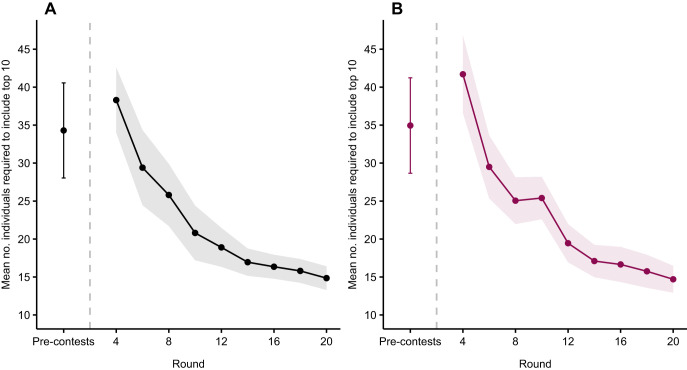
**Mean number of individuals needed to include the true top 10 in simulated contests.** ‘Pre-contests’ refers to selection based on trait ranks only for (A) males and (B) females. Rankings across rounds were estimated using Bradley–Terry models with cumulative contest data. Points, error bars and shading represent means±95% confidence intervals across 20 replicate simulations, each with 100 simulated crayfish.

On average, 34.3 males (95% confidence interval, CI [28, 40.6]) and 35 females (95% CI [28.7, 41.2]) were required to include the top 10% of individuals based on pre-contest trait ranks (Fig. 1). Using contest data, the number of individuals required decreased non-linearly with the number of contest rounds for both males (*P*<0.001; Table 2) and females (*P*<0.001; Table 2). After 4 rounds, an average of 38.3 males (95% CI [34, 42.6]) and 41.7 females (95% CI [36.6, 46.8]) had to be selected to guarantee that the top 10 were included. This number decreased with additional rounds, reaching 20.8 males (95% CI [17.2, 24.4]) and 25.4 females (95% CI [22.6, 28.2]) after 10 rounds, and plateaued at approximately 17 individuals for both sexes after 14 rounds.

**Table 2.** Summary of six generalised additive models (GAMs) predicting the number of individuals required to ensure the top 10% are included, with either round or interaction between α and round

Model	Smooth term	e.d.f.	*F*	*P*	Deviance explained (%)
Male (Round)	s(Round)	7.71	122	<0.001	84.7
Female (Round)	s(Round)	7.14	115	<0.001	83.2
Male hybrid (100)	te(Alpha, Round)	17.6	37.8	<0.001	32.2
Female hybrid (100)	te(Alpha, Round)	20.6	37.6	<0.001	35.6
Male hybrid (40)	te(Alpha, Round)	18.9	62.3	<0.001	45.2
Female hybrid (40)	te(Alpha, Round)	19.8	54.1	<0.001	43.3

EDF, effective degrees of freedom.

### Selection based on hybrid scores

**Fig. 2.**
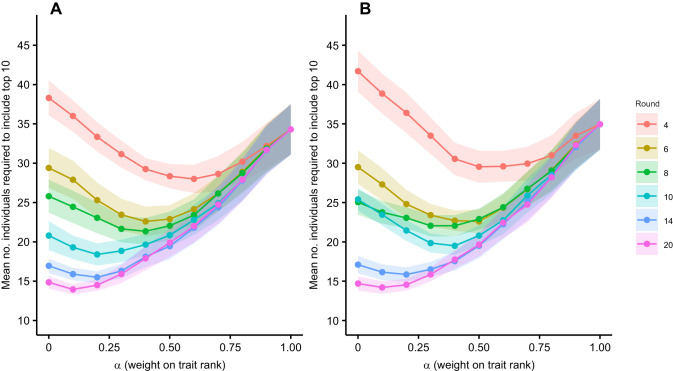
**Influence of α (weight on trait rank) on mean number of *Cherax* *destructor* individuals required to ensure inclusion of the top 10 performers (100 individuals per group).** α determines weighting between trait-based (α=1) and contest-based rankings (α=0). Results are shown for (A) males and (B) females for a subset of rounds. Points and shading represent means±s.e.m. across 20 replicates, each with 100 simulated crayfish.

When contests involved all 100 individuals, the number of males required to ensure inclusion of the top 10% depended on a non-linear interaction between the weighting of trait ranks versus contest ranks (α) and the number of rounds (*P*<0.001), with the model explaining 32.2% of the deviance (Table 2, Fig. 2A). This interaction indicates that the α value associated with the smallest number of individuals that need to be included decreased as the number of rounds increased. For example, at round 4, the optimal α was 0.6, requiring selection of 28 males on average (95% CI [24.5, 31.5]), whereas by round 20, the optimal α decreased to 0.1, requiring only 14 males (95% CI [12.7, 15.2]).

Females exhibited a similar pattern, where the number of individuals needed depended on a non-linear interaction between α and the number of rounds (*P*<0.001; Table 2, Fig. 2B). The GAM explained 35.6% of the deviance (Table 2). At round 4, the optimal α was 0.5, where 29.6 females needed to be selected to ensure the top 10 were included (95% CI [25.5, 33.6]). By round 20, the optimal α decreased to 0.1, where only 14.2 females, on average, needed to be selected (95% CI [12.7, 15.7]).

**Fig. 3.**
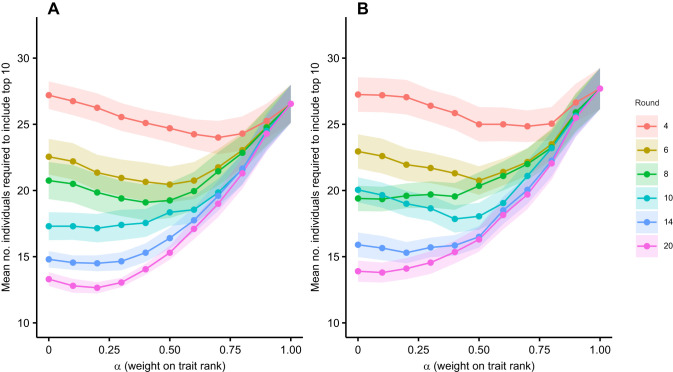
**Influence of α (weight on trait rank) on mean number of individuals required to ensure inclusion of the top 10 performers (top 40 trait rank).** The bottom 60 individuals were excluded prior to contests based on trait rank. α determines weighting between trait-based (α=1) and contest-based rankings (α=0). Results are shown for (A) males and (B) females for a subset of rounds. Points and shading represent means±s.e.m. across 20 replicates, each with 40 simulated crayfish.

When contests involved only the top 40 individuals based on trait ranks, the relationship between α and the number of rounds was still non-linear for males and females (both *P*<0.001; Table 2). For males, the optimal α decreased from 0.7 at round 4 to 0.2 by round 20, reducing the number of individuals needed to include the top 10 from 24 (95% CI [21.5, 26.5]) to 12.7 (95% CI [11.8, 13.5]) (Fig. 3A). Similarly, for females, the optimal α decreased from 0.7 to 0.1 from round 4 to round 20, with the number required declining from 24.9 (95% CI [22.5, 27.2]) to 13.8 females (95% CI [12.3, 15.3]; Fig. 3B). Optimal α values were slightly higher when contests were restricted to the top 40 individuals compared with when all 100 individuals competed.

### Empirical trait and contest data

**Fig. 4.**
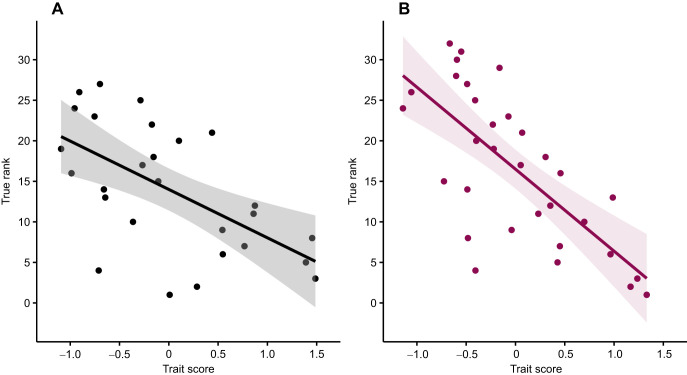
**Relationship between trait score and true rank for individuals*.*** For (A) males (*n*=27), trait score was calculated as equal weighting of claw size and claw strength, and for (B) females (*n*=32) as equal weighting of body length and claw strength. True rank was estimated from a Bradley–Terry model after 11 rounds of contests, and lower ranks indicate better performance. Shading represents standard error.

Males with higher trait scores, reflecting larger claw sizes and greater claw strength, had better (lower score) contest rankings (*R*^2^=0.35, *F*_1,25_=23.23, *P*=0.001; Fig. 4A). Similarly, females with higher trait scores, and thus larger body lengths and greater claw strength, tended to exhibit better rankings (*R*^2^=0.51, *F*_1,30_=31.54, *P<*0.001; Fig. 4B).

When determining the actual contribution of each trait to dominance, claw size was more important in predicting contest rank in males (estimate=−4.21, *t*=−3.23, *P*=0.004) than claw strength (estimate=−1.75, *t*=−1.34, *P*=0.19). However, increases in both traits were still associated with better rankings (*R*^2^=0.38, *F*_2,24_=7.46, *P*=0.003). For females, body length was a stronger predictor of final rank (estimate=−6.65, *t*=−5.83, *P*<0.001) than claw strength (estimate=−3.46, *t*=−3.03, *P*=0.005), with increases in both traits contributing to better overall rankings (*R*^2^=0.57, *F*_2,29_=19.8, *P<*0.001).

**Fig. 5.**
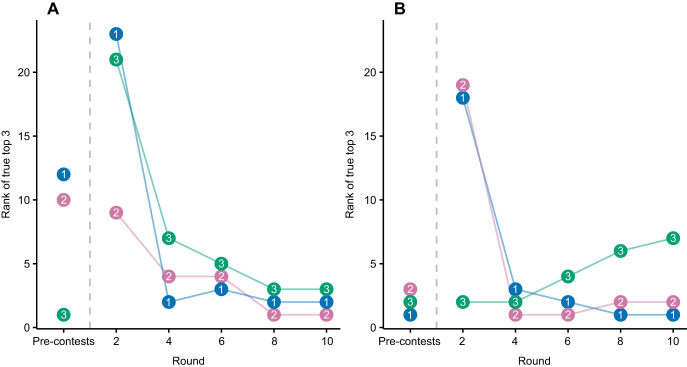
**Rank of top three *C. destructor* in staged contests across rounds.** For (A) males and (B) females, the number in data points represents the true rank of the individual (1, 2 and 3), assumed as their estimated rank after 11 rounds of contests. ‘Pre-contests’ represents rank based on trait scores. Contest ranks were estimated at each round using a Bradley–Terry model.

Based on trait ranks alone, 12 males and 3 females needed to be selected to ensure inclusion of the top 3 (10%) individuals (Fig. 5). As the number of rounds progressed, the number of individuals required decreased. After 2 rounds, 23 males and 19 females had to be selected to include the top 3, which decreased to 3 males and 7 females after 10 rounds.

## DISCUSSION

This study aimed to test whether approaches common in ecology could be used to test the efficacy of talent identification strategies using crayfish contests as a simplified and tractable system. Our results demonstrate that by combining trait-based predictions with repeated contest outcomes, one can effectively narrow the pool of individuals needed to identify the top performers. In both simulated and empirical analyses, the number of individuals required to capture the top 10% of dominant performers declined non-linearly across contest rounds. For example, in the full simulated population, selection based solely on pre-contest trait rankings required an average of 34 males and 35 females. After 10 rounds of contests, this number dropped to 21 males and 25 females and eventually plateaued at around 17 individuals per sex by round 14. Hybrid models that integrated trait and contest data were even more efficient. When contest participation was restricted to the top 40 individuals based on trait ranks, which mimics pre-selection processes in sports, efficiency improved further, requiring only 13 males and 14 females by round 20 to capture the top 10%. Empirical data based on male and female *C. destructor* supported these findings. For males, trait scores (claw size and strength) explained 35% of the variation in contest rankings, while for females, trait scores (body length and claw strength) explained 51%. In early rounds, identifying the top 3 performers required selecting 23 males and 19 females; by round 10, this dropped to just 3 males and 7 females. These findings highlight how trait-based pre-selection, refined by contest outcomes, can be used to efficiently identify high-performing individuals even under uncertainty.

Our experiment and analyses serve as a proof of concept for the broader aims of applying ecological approaches to testing talent identification strategies. Our findings offer valuable insights that form the foundation from which to develop improved statistical models that better capture the relative and potentially interactive effects of traits on dominance. Future directions include staging contests in more complex environmental situations such as using multiple competitors and environmental variation, which in a sporting context would resemble multi-player game situations and different weather and field conditions. Additionally, we made simplifying assumptions such as using only a limited set of traits to predict dominance outcomes. It would be very interesting to determine whether additional traits can improve contest outcomes and how different traits are ranked. A trait-ranking approach would test whether different traits contributed equally and additively to performance and could thereby facilitate predictions of individual fitness in particular natural environments, or game performance in a sport context. Future experiments can incorporate these more refined trait characteristics into statistical models and test their predictive power across multiple independent groups. Ecologically, extending this work into mesocosm experiments where success can be defined by survival, growth and population sizes will further increase its relevance to nature and enhance analogies to the complexity of sports. Such ecological systems would offer a closer parallel to the multi-agent, dynamic conditions of team sports, where individuals interact in complex, unpredictable environments.

Identifying true potential in youth athletes requires navigating a complex landscape where age, size and developmental maturity can obscure underlying future quality, but ecological model systems may also help to address this issue. In youth soccer, players are typically grouped into annual age cohorts that include substantial variation in both chronological age and physical maturity, which makes the identification of future potential even more complex (Carling et al., 2009; Gil et al., 2014). For example, in an under-13 elite soccer squad, a player born in January can be 10% older than a player born in December. This discrepancy confers significant advantages in physical performance and coordination, leading to a widespread bias where older, more mature players within cohorts are often mistaken for more promising individuals (Gastin and Bennett, 2014; Helsen et al., 1998; Philippaerts et al., 2006). The stronger and more physically developed individuals are better at the time of selection but this may have little relevance to their actual relative performance at maturity if later-maturing individuals are also provided with equivalent opportunities. Consequently, soccer academies around the world are disproportionately filled with players born in the early months of the year (Figueiredo et al., 2009) – up to 60% of players in elite academies may be born in the first 3 months of a cohort year (Hunter et al., 2023). This phenomenon, known as the relative age effect, represents not only a form of structural discrimination but also a missed opportunity for club success, as many fail to identify younger players with long-term potential. Bio-banding is one attempt to mediate the effects of age-biases in team sports where players are placed into ‘bands’ or groups based on stages of physical development rather than age for specific competitions and training (Bradley et al., 2019; Lüdin et al., 2022; Malina et al., 2019). Despite the positive attitude towards these measures, there is limited evidence that selection processes or outcomes have changed as a result of bio-banding, because this banding usually occurs after players have been selected for talent squads (Bradley et al., 2019; Lüdin et al., 2022; Malina et al., 2019). An alternative approach to addressing biases in youth selection is to develop and use age- and size-corrected assessments of individual players, particularly for skill-based traits because these are less sensitive to rapid changes in growth and physical development (Hunter et al., 2022). Incorporating age- and size-corrected traits into sequential selection strategies and then testing their efficacy will be complicated, but analyses such as ours based on ecological model systems such as crayfish may provide novel and easily manipulated systems. Outcomes of competitive interactions in these ecological systems depend on multiple overlapping variables such as age, size, aggression and ecological context (Graham and Angilletta, 2020; Gruber et al., 2016; Walter et al., 2011). Therefore, ecological systems offer a unique opportunity to examine how performance traits interact under realistic but experimentally tractable settings. For example, researchers can disentangle the confounding effects of developmental timing and physical growth using experimental manipulations, and then test different strategies for assessing relative performance under controlled conditions. Insights from such experiments could inform more equitable, evidence-based approaches to youth talent identification, ultimately helping clubs recognise and develop players based on true potential rather than temporary physical advantages.

While this study has focused on the value of ecological perspectives and systems for improving talent identification frameworks for sport, the conceptual exchange need not be unidirectional. The analytical tools and decision-making frameworks developed for identifying top performers in sport may hold reciprocal value for ecological applications, particularly in conservation programmes involving animal translocations or captive breeding for reintroduction. These initiatives often hinge on selecting individuals with the greatest likelihood of long-term survival and reproductive success in the wild (Berger-Tal et al., 2020; Seddon et al., 2007). For instance, Biggins et al. (2011) examined the reintroduction of black-footed ferrets (*Mustela nigripes*) into a previously unoccupied prairie dog colony and found that wild-born individuals, compared with captive-born counterparts, exhibited significantly higher short-term survival. The captive-born ferrets showed greater nightly movements that were correlated with lower survival (Biggins et al., 2011). These findings underscore the influence of behavioural traits, such as boldness, shelter use and competitive ability, on reintroduction outcomes. In this context, a refined understanding of trait-to-performance relationships and its connection with success, as found in talent identification strategies in sport, could significantly enhance selection accuracy for specific favourable traits. By applying such frameworks, ecologists may improve the efficacy of translocation and reintroduction efforts by identifying and prioritising individuals with favourable trait combinations most aligned with post-release success, especially during the high-risk early stages. Beyond conservation, this approach is also relevant to understanding how whole-organism performance translates into competitive success and ultimately fitness. By explicitly linking morphological and behavioural traits to performance outcomes and success, this framework provides a tool to revisit fundamental questions within the morphology–performance–fitness paradigm (Arnold, 1983). More broadly, this cross-disciplinary approach highlights a fertile area for innovation, where sports science and ecology co-develop tools for managing individual variation in complex, high-stakes environments.

The cross-disciplinary approach used in this study shows promise not only for improving fairness and accuracy in youth sport selection but also for ecological applications such as translocation and reintroduction programmes, where choosing the right individuals may be critical.

## Supplementary Material

10.1242/jexbio.251395_sup1 Supplementary information
