# Supplementary material for: Applied performance ecology: testing strategies of talent identification in sports using ecological systems
Source: J Exp Biol. 2026 Apr 7;229(7):jeb251395. doi: 10.1242/jeb.251395 (PMC13091497; doi:10.1242/jeb.251395)
Supplement: Supplementary information [file jexbio-229-251395-s1.pdf]

Supplementary Materials and Methods

Equations for male simulation:

$$ClawSize_i = 0.9961 \times BodyLength_i + \varepsilon_1,\ where\ \varepsilon_1 = N(0, 0.2601^2) \text{ (S1)}$$

$$ClawStrength_i = 0.8449 \times ClawSize_i + \varepsilon_2, where\ \varepsilon_2 = N(0, 0.5388^2) \text{ (S2)}$$

$$TraitScore_i = 0.5 \times ClawSize_i + 0.5 \times ClawStrength_i \text{ (S3)}$$

$$Dominance_i = TraitScore_i + \varepsilon_3, where\ \varepsilon_3 = N(0, 0.7^2) \text{ (S4)}$$

Equations for female simulation:

$$ClawSize_j = 0.9761 \times BodyLength_j + \varepsilon_4,\ where\ \varepsilon_4 = N(0, 0.2186^2) \text{ (S5)}$$

$$ClawStrength_j = 0.7264 \times ClawSize_j + \varepsilon_5, where\ \varepsilon_5 = N(0, 0.692^2) \text{ (S6)}$$

$$TraitScore_j = 0.5 \times BodyLength_j + 0.5 \times ClawStrength_j \text{ (S7)}$$

$$Dominance_j = TraitScore_j + \varepsilon_6, where\ \varepsilon_6 = N(0, 0.7^2) \text{ (S8)}$$

Journal of Experimental Biology • Supplementary information

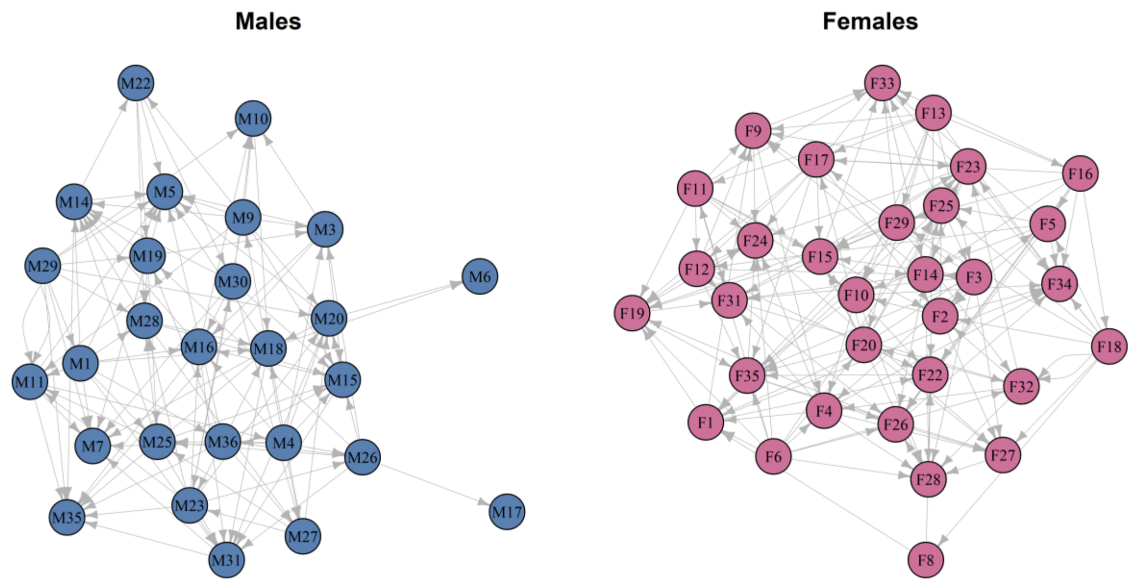

Fig. S1. Contest interaction networks for male (n = 27) and female (n = 32) *Cherax destructor* across 11 rounds. Arrows are directed towards the loser of the contest.

Journal of Experimental Biology • Supplementary information
